# In reply: the improvement technique for reproducibility of diffusion tensor image analysis along the perivascular space (DTI-ALPS) for evaluating interstitial fluid diffusivity and glymphatic function

**DOI:** 10.1007/s11604-023-01431-0

**Published:** 2023-04-20

**Authors:** Toshiaki Taoka

**Affiliations:** Department of Innovative Biomedical Visualization (iBMV), Nagoya University Graduate School of Medicine, 65 Tsurumai-Cho, Showa-Ku, Nagoya, Aichi, 466-8550 Japan

**Keywords:** Glymphatic system, DTI-ALPS, Brain interstitial fluid dynamics, Reproducibility, Harmonization

Dear Dr. Yuya Saito,

Thank you for your interest in our paper, entitled “Reproducibility of diffusion tensor image analysis along the perivascular space (DTI-ALPS) for evaluating interstitial fluid diffusivity and glymphatic function: CHanges in Alps index on Multiple conditiON acquIsition eXperiment (CHAMONIX) study” [1]. This paper demonstrated that imaging conditions, such as imaging sequence and head position, affect the ALPS-index when applying the ALPS method, and the importance of unifying the imaging conditions. We emphasized that comparing ALPS indexes from different imaging sequences does not make sense, and that efforts should be made to match conditions as much as possible at the time of acquisition of the images.

I read your letter with interest. Harmonization techniques are known to minimize differences between data sets and obtain reliable results when multiple data sets are integrated for analysis, for example, with different scanners [2]. In order to integrate different data sets and obtain reliable results, it is necessary to select and integrate appropriate methods for each data set, which requires a considerably higher level of skill and knowledge [3, 4]. I understand that harmonization techniques are generally not easy for newcomers to perform. In this regard, I believe that harmonization techniques are rather particularly useful for the purpose of effectively utilizing large existing data sets. That is because the imaging conditions of an existing data set can no longer be changed.

In prospective studies, efforts to align imaging conditions and head position should still be important. I do not believe that imaging without the right conditions will yield good results just because harmonization technology is available. Especially when the number of samples is small, uniformity of imaging conditions is essential to obtain meaningful data. It is fundamental to scientific measurement to do one's best when acquiring data. I consider that harmonization techniques should not be an exemption for poor-quality data collection.

It is hoped that harmonization techniques will allow for greater use of existing data and provide useful data from previous cohort studies. I hope that you will promote the harmonization technology widely in the near future. It would be nice if you could provide an environment that many people can use. We are pleased that our work on the reproducibility of the ALPS method has motivated your work on harmonization.
